# Filling Teeth—Some Special Points to Be Observed

**Published:** 1880-12

**Authors:** E. Noyes

**Affiliations:** Chicago.


AMERICAN JOURNAL

OF

DENTAL SCIENCE.

Vol. XIV. THIRD SERIES—DECEMBER, 1880. No. 8.

### ARTICLE I.

### *Filling Teeth—Some Special Points to be Observed.*

BY E. NOYES, D. D. S. CHICAGO.

There are many other things comprehended in the science and art of dentistry, as practiced nowadays, besides filling teeth, and such things as chemistry, anatomy and physiology, pathology, therapeutics, and the knowledge and management of all the special diseases that require consideration or treatment by the dentist, are so comprehensive in their scope, so various and intricate in their details and so difficult of thorough mastery, that a very large proportion of the *student's* time must necessarily be given to *them*. He may receive the impression that in actual practice he is to find these various subjects claiming his attention in much the same relative proportions. He will probably find, in *fact*, that three-fourths, and perhaps, nine-tenths, of his time devoted to patients having natural teeth, will be occupied with the operations of fillings; while some other acquirements, which perhaps consumed much more of his time and effort while a student, are called into direct exercise but seldom. It is not a proper inference from this that a student should be required to spend most of his time in practicing the operations of filling. He only needs to spend enough time, and under competent instruction and supervision, to acquire correct *habits* of manipulation and a good understanding of the mechanical and artistic principles involved; then if we have such a degree of manipulative aptitude and ingenuity, and of artistic perception as every dentist ought to be endowed with by nature, he will soon become expert in these operations that he must be performing all the time, while the things that he is called upon to do but very seldom must be thoroughly mastered and practiced while a student, if they are ever to be.

The operations of filling teeth are mechanical, and the principles and relations *directly* involved are mechanical and artistic ones. They may be performed with little or no reference to any others, as though working upon a dead body instead of a living person. The man who does so may be a fine, and even artistic *mechanic*, but has little claim to be called a *professional* man.

The principles and relations indirectly involved in consequence of the vitality of the organs to be operated upon, and the mutual relations and dependences between them and the living body in health and disease are recognized and understood so as to guide or modify, or control the operations, in proportion as a man's education and ability enable him to rise above the level of the mechanic to that of the professional man.

So large a proportion of time being spent in the various operations connected with filling teeth, it follows, of course, that success and usefulness *very largely* depended upon the ability and disposition to make thoroughly good fillings. Dentists are entirely isolated from each other during hours, and have few and scanty opportunities to observe each other's methods, or even to talk together about them, and though there is a great variety and a wide range of differences, in the different operations, and the different patients, so that we may say that no two operations are *exactly* alike, yet *practically* we have, after a time, a continual repetition of things we have done before. This makes it inevitable that we shall acquire, after a while, some very firmly fixed *habits* of operating, and get to running in some pretty deep *ruts*. There may be no great objection to this if the *habits* are only good ones and the *ruts* are smooth and free, so as to facilitate easy traveling, but no man has the best methods of doing *all* things, and we cannot be too earnest in endeavors to observe and compare the methods of other men with our own, for the purpose of finding out whether some of them may not be so much better than ours as to justify the trouble of unlearning old ways and acquiring new ones. Most of us are workers and not talkers, and ought to make the special effort necessary to be able to describe an operation minutely and accurately, so that those listening may have in their imaginations an image or picture similar to the one in the mind of the narrator, and not, as is often the case, a very different one. If it were possible, it would prove of the greatest benefit for each of us to become familiar with the habits and methods of a dozen or twenty of the best operators we could find, as a student does with his preceptor. The clinics and discussions at society meetings should be made to all that is possible toward that end.

In filling teeth all points are *special*—that is to say, each step in every operation requires the best attention and most perfect performance that we are able to give it, and some things not mentioned in this paper may be of as much importance as anything in it. When a mouth is presented for filling teeth, it is often desirable, and in some cases indispensable, to clean the teeth before anything else is attempted, and I feel moved to speak just here in emphatic condemnation of the inexcusable negligence of very many dentists in regard to the removal of tartar. It is a burning shame and almost a criminal neglect for any man to dismiss a patient with the implication that nothing more needs to be done, until he has thoroughly removed every particle of tartar that can be removed (unless the teeth were free from it before.) The disagreeableness, difficulty and the small compensation attending this operation are not sufficient excuses for the common neglect to perform it, and very often the service that can be rendered in this way, accompanied by specific instructions in regard to future management in respect to cleanliness, *may* prove of greater service to our patients than all the fillings we may make for them. I have seen the condition and appearance of some mouths totally changed—revolutionized, as a consequence of such treatment and instructions.

The next important step, preliminary to operations of filling, is to make a very careful examination of the mouth, going through it with floss-silk, mouth-glass and small excavators or exploring instruments, tooth by tooth, till the condition of each one is ascertained as nearly as possible with reference to decay, and a permanent record made in a book to be kept for the purpose. Similar cases can be used as in the recording of operations. Doubtful places should be indicated and afterward separated one by one with rubber, or otherwise, for perfect examination with the record book will at any time show what has been done and what remains to do.

The advantages of such an examination are very many. The most important, perhaps, is the opportunity it gives to plan the entire series of operations and arrange the various items so as to be consistent with each other as well as suitable in each instance. The saving of time in frequent re-examinations, and security from the liability to overlook or forget important operations, are of great consequence also. It is a very poor service that a man renders his patients if he only fills cavities that are pointed out to him, without taking much care or responsibility for the entire mouth, and almost all our patients ought to receive just such thorough and comprehensive care and treatment, whether they come in the first place with any such desire and expectation or not. Most of them will be glad to allow it if properly advised, and treated with frankness and sincerity.

The sequence of operations can often be more conveniently arranged if the whole series are presented to the eye at once upon a suitable diagram. It also gives the best opportunity that can be had for estimating the probable cost in the cases when it is necessary or desirable to do so. There are very many people who are unwilling to begin a series of dental operations, as they are obliged to a severe illness, without the remotest idea whether the expense will be nearest to $25 or $500, and there is not often *very* serious objections to giving patients about as much information as we can obtain beforehand ourselves. It will seldom be definite enough to be of much use for "shopping," and of course there is little motive to give prices to those who want them only for comparison with those of other dentists, but those whose circumstances make it really desirable that they should know something about what they will have to pay can usually be told within, say, 25 per cent, the margin varying greatly in different cases. It is not well to do much *guessing*, else we shall get *left* ourselves with too small a price, or our patient will leave for fear of one too large.

Having made the examination and formed the general plan of operations, the next step is to obtain the proper opportunity to work.

This, in respect to grinding surface cavities and buccal cavities consists in little else than the adjustment and retention of the rubber-dam. Patients usually regard the clamp as a barbarous instrument, and its use ought probably to be confined to buccal cavities in the back part of the mouth, and in lower teeth. In these it keeps the rubber from falling against the cavity, giving room to work, and does not strain the corner of the mouth so much as an instrument with a handle, held against the tooth by an assistant. In all the teeth forward of the molars the crescent-shaped instrument held against one side of the tooth is far preferable to the clamp for exposing cavities at the margin of the gum. In all grinding surface cases when necessary, and in proximal ones, when the rubber is liable to slip off from one or both of the two teeth to be covered, the most useful auxiliary that I know is a string of waxed floss with two beads tied upon it. This is quickly and easily tied around almost any tooth (of course *before* the rubber is attempted to be put on,) and its application is seldom more than slightly painful, if at all. It can usually be done even for very small children, and the rubber once slipped over them is in no danger of coming off. In some cases where the adjoining teeth are very close, and they are not needed for *retention*, time can be saved in *adjustment* by tying on the beads, by stretching the rubber down over them on each side, the edges of the hole are held in proper position for readily drawing down between the teeth with the waxed floss. If your assistant is used to it and the mouth is large enough for two pairs of hands to work in, it can be done quicker by one holding the rubber while the other forces it in between the teeth. These appliances, and the orange wood or hickory wedge, for crowding back and retention above the margins of approximal cavities are about all that I have ever found necessary for retaining the rubber dam in any place where I desire to use it. Many small grinding surface cavities in second upper molars and wisdom teeth can be entirely filled in less time than would be required to adjust a rubber dam over them. If a good sized piece of spunk is placed upon the duct of steno, and a napkin laid on a piece of rubber carried into the cheek around behind the last tooth, the finger that holds it in place resting usually on the spunk that covers the duct, the rubber lining will keep the napkin from becoming much wet for a good while. If such cavities are large enough to require a lining of oxychloride of zinc, the rubber should be adjusted upon them in the usual way at almost any expense of time and trouble, *especially if the filling is to be amalgam.* I believe that material is more damaged by a little water than any other with which I am acquainted, and the water is less certain to provide its own remedy by stopping the progress of the operation.

Obtaining the proper opportunity to work in the case of most approxinal cavities consists first in the previous separation of the teeth by means of wood, or rubber, or cotton wedges, and afterward in driving a firm wedge of orange wood or hickory between the teeth close to the gum after the rubber is in place. Those who have not tried it often will be surprised to find how very few cases there are in which it cannot be forced high (or low) enough to expose the margins of cavities extending far under the gum. It will often have to be made thin and bent up (or down) like a bow and carried very near or quite to the process. Patients are very apt, and probably justly, to call these wedges barbarous as well as the clamps, but their great and indispensable usefulness and the lack of any substitute for them, have kept me thus far in the constant use of them. The *previous* separation I regard as of *very* great usefulness and importance in most cases, and I see very many teeth which in my judgment have been injured by the cutting which the want of it made necessary—many front teeth that have been inexcusably mutilated, unnecessarily injuring their appearance beyond remedy. Teeth that have space between them at the necks and touch near the grinding surfaces in a full or crowded arch, so that if cut they will come together and touch again, are often made more certain to decay the second time than the first, by such treatment, the surfaces in contact being less convex and the necks nearer together than before. These considerations are of most importance of course in the cases which do not require cutting from the grinding surface, but they have nearly equal weight when they come to the grinding surface by a small notch, and do not involve a *very* large proportion of the approximal surface. This previous separation is a great deal of trouble, both to the dentist and his patients, and it is only reluctantly, and by experience and observation of its great value, that any one is likely to form the habit of doing it in all the cases in which it is desirable.

I believe myself that it is well to fill a great many small cavities with soft foil, wholly or in part, and to use a great deal of foil without annealing in all cavities which do not require that the finished plug should be a perfectly coherent mass. When cohesive foil is added to soft foil, care should be taken to drive the first portions into the soft foil like a wedge, and not plaster them over the surface, as would be done if the previous mass were cohesive.

A special point about finishing fillings, and one often neglected, is to cut away *enough* of the gold, so that there will be no thin proportions left overlapping the margins (this applies to both approximal and grinding surface fillings,) and it sometimes requires very close scrutiny to determine just when all the overlapping gold is removed, and it cannot be properly done at all unless the border of the cavity has been well prepared in the first place. Then, of course, the work should be left smooth and well polished, if an approximal filling; in grinding surface, if smooth and not overlapping the margins, a *polish* is of little consequence.

Contour fillings, especially corners and sides of front teeth, need very careful attention as to their shape, and the exercise of good taste and a good eye for form. A great many such fillings are greatly exaggerated in size and others are too full in some parts and not full enough in others. I have seen many that were fuller than the original form of the tooth, seeming to say to the eye, "This work is a *little* better than Nature's was. I will therefore show a little more of it and make it generally more conspicuous." Good taste requires that such operations should please the eye by a close reproduction of the original form, a little less than the original in bulk, being of conspicuous color, and at best but an artificial substitute for nature, which should thrust itself upon the attention as little as possible.

Another special point is a retaining point, and which I believe is, in many cases, made too deep and too near the margin, especially if at the cervical wall. The only use of a retaining point deep enough to set a screw in is to have a screw set into it. The principal office of a retaining point or a groove is to hold in place the first pieces of gold packed into it. If they will do that they are usually sufficient for all necessary purposes as dovetails to retain the finished filling. At the necks of the teeth they must often be made with a very small drill, often much smaller than White's No. 1, and the grooves with a *very narrow* chisel and is seldom necessary to cut them deeper than their diameter.

Large cavities, more especially approximal ones in bicuspids and molars, are sometimes left with too deep natural undercuts, and the overhang too frail. Do not be afraid to get a saucer shaped cavity that looks as if a filling would not stay in it, as a consequence of cutting away all the weak overhanging portions of enamel that are reasonably sure to break down and split off after a while if left there. The buccal and palatal walls are especially liable to fail if left too thin and weak. On the other hand, the arch of enamel from the buccal to the palatal walls at the approximal border of the grinding surface, should be sacrificed with reluctance, especially if the cusps are long and the sulci between them deep. It should be retained if there is any probability that it will stand. Undercuts and retaining points, to be of much value or permanence, should be made wholly in dentine and not in enamel.

I think I must have a little to say about amalgam, a material that has been much abused; perhaps the worst abused by some of those who use the most of it. It is my opinion that the proper use of amalgam calls for a higher degree of fidelity and carefulness, and an equal degree of skill and manipulative ability as any material now in use for fillings, but it is very easy to make them so that they will appear well at the time, and yet be so very imperfect as to be in the end of very little use for the preservation of the teeth. There are some kinds of amalgam in common use with which I believe it a physical impossibility to make a good filling, and there are many with the qualities of which I am not acquainted. The same one should be used until perfectly familiar with its peculiarities, so as to be master of its working qualities, for they will not all admit of the same style of manipulation. The cavities must be very carefully prepared, and in almost every instance the rubber-dam *must* be used, for there must be no *chance* of getting moist, unless in the forlorn cases where a *good* operation is out of the question. The materials should be quickly and thoroughly mixed in just the right proportions, and will be so dry and powdery as to be a nuisance to get where it is wanted in many cases. It must be *packed* in small quantities and with points that will condense it well into corners and undercuts (a small ball burnisher is best in places where it will go.) It sets so fast that two mixings will often be necessary in places where it is difficult to carry it to its place and the filling is large. It must be packed and worked together solidly throughout, with much the same force as is used for condensing by hand the surface of a soft foil filling. If dry, as it should be, it is very difficult to build up a corner with it, and in most compound fillings a matrix should be used. One is easily made by bending slightly, if necessary, a piece of separating file and pushing in a wedge of soft wood behind it, or a piece of sheet lead may be bent around one side of the tooth across the cavity and held by ligatures. Twice the time will be saved that it takes to adjust them and better work can be done and a more satisfactory contour obtained. They can be immediately removed and the filling trimmed to very nearly its final form. It is indispensably necessary that every amalgam filling should be polished at a sitting subsequent to its insertion.

It is impossible to deny that amalgam fillings *can* be made in the way I have indicated so as to be thoroughly good operations, in the same sense and for the same reasons that well made gold fillings are good operations. The characteristics that make them so may be described in the same terms, that is, they will not leak; they will take and retain a smooth surface; they will make a smooth margin that will not crumble. The difficulty of doing good work with amalgam often increases with the smallness of the cavities if approximal ones, and I believe that most approximal cavities not reaching the grinding surface, if of sufficient depth and having good walls, are better filled with tin foil than amalgam. If Williams' tin cylinders are used, of style B, which are rolled pretty hard, a little practice will enable one to make them in many cases as quickly and easily as with amalgam, and with the advantage of finishing at once. There are many places, especially in young or soft, teeth in which I question whether gold will make as useful a filling as tin. The range of applition for tin is quite limited, but within its proper limits I believe it one of the most useful materials we have. The great convenience of these tin cylinders, and the fact that they appear to be but little known or used, are perhaps sufficient reasons for directing your attention to them particularly. The tightly rolled ones, style 1 B, and 2 B, can be used in about the same way as folded pellets of foil, and by spliting them lengthwise with the scissors as small pieces as are desired can be easily obtained. Tin is practically non-cohesive, and one who never makes a gold filling without lighting his annealing lamp, will be likely to deed a little practice before acquiring facility in its use. Every piece, even to the very last, must go deeply enough into the cavity to be retained by wedging, but it is considerably softer and more easily wedged tight than gold; the surface condensation will take effect more deeply, and it is far more rapidly and easily trimmed and finished.

I am not one of those who desire to see the use of amalgam increased, but I wish that men should feel as much under obligation to maintain a high standard in their operations with it as they do in their gold operations, and to keep them as carefully under the supervision of their consciences and their professional pride. If that is accomplished, amalgam fillings will be found less remunerative than gold ones, and so the motives of self-interest will be on the side of the better material. I believe that such treatment of the "amalgam question" will do more toward restraining and diminishing its improper use than the wholesale and unaeasonable denunciation that we sometimes hear.—*Ill. State Dent. Society Trans.*

